# Design of, and enrollment in, the palliative care communication research initiative: a direct-observation cohort study

**DOI:** 10.1186/s12904-015-0037-8

**Published:** 2015-08-19

**Authors:** Robert Gramling, Elizabeth Gajary-Coots, Susan Stanek, Nathalie Dougoud, Heather Pyke, Marie Thomas, Jenica Cimino, Mechelle Sanders, Stewart C. Alexander, Ronald Epstein, Kevin Fiscella, David Gramling, Susan Ladwig, Wendy Anderson, Stephen Pantilat, Sally A. Norton

**Affiliations:** 1 Family Medicine Research Programs, University of Rochester, 1381 South Avenue, Rochester, NY 14620 USA; 2 School of Nursing, University of Rochester, 601 Elmwood Avenue, Box SON, Rochester, NY 14642 USA; 3 Division of Hospital Medicine and Palliative Care Program, University of California, Clinical Sciences Building Suite C-126, 521 Parnassus Ave, Box 0131, San Francisco, CA 94143-0131 USA; 4 Purdue University, Matthews Hall, 812 West State Street, West Lafayette, IN 47907-2060 USA; 5 University of Arizona, 301 Learning Services Building, 1512 East First St., Tucson, AZ 85721 USA; 6 Palliative Care Program, University of Rochester Medical Center, 601 Elmwood Avenue, Box 687, Rochester, NY 14642 USA

**Keywords:** Epidemiology, Recruitment, Enrollment, Communication, Prognosis

## Abstract

**Background:**

Understanding the characteristics of communication that foster patient-centered outcomes amid serious illness are essential for the science of palliative care. However, epidemiological cohort studies that directly observe clinical conversations can be challenging to conduct in the natural setting. We describe the successful enrollment, observation and data collection methods of the ongoing Palliative Care Communication Research Initiative (PCCRI).

**Methods:**

The PCCRI is a multi-site cohort study of naturally occurring inpatient palliative care consultations. The 6-month cohort data includes directly observed and audio-recorded palliative care consultations (up to first 3 visits); patient/proxy/clinician self-report questionnaires both before and the day after consultation; post-consultation in-depth interviews; and medical/administrative records.

**Results:**

One hundred fourteen patients or their proxies enrolled in PCCRI during Enrollment Year One (of Three). Seventy percent of eligible patients/proxies were invited to hear about a communication research study (188/269); 60 % of them ultimately enrolled in the PCCRI (114/188), resulting in a 42 % sampling proportion (114/269 eligible). All PC clinicians at study sites were invited to participate; all 45 participated.

**Conclusions:**

Epidemiologic study of patient-family-clinician communication in palliative care settings is feasible and acceptable to patients, proxies and clinicians. We detail the successful PCCRI methods for enrollment, direct observation and data collection for this complex “field” environment.

## Background

*Our findings suggest that the most fundamental medical choice patients with [serious illness] face—the decision between life-extending therapy and comfort care—may be highly influenced by their understanding of their prognoses* [1]*.* –SUPPORT Study, 1996

Communicating about prognosis is important for decision-making in serious illness [2–12]. However, prognosis discussions can be daunting for physicians, patients and families [13–16], resulting in patterns of prognostic avoidance or obfuscation in the usual care of seriously ill patients [14, 17–19]. Indeed, these endemic norms makes studying prognosis communication in the natural setting quite challenging—resulting in nearly no empirical data about the characteristics of prognosis conversations that promote high quality treatment decisions and better quality of life for patients with advanced cancer.

Palliative care is grounded in fostering high quality communication amid serious illness. Prognosis conversations are a fundamental feature of palliative care consultations [20–22] and emerging evidence suggests that these discussions are a key ingredient [21–24] in palliative care’s benefit to persons with advanced cancer [25–27]. Little is known, however, about how patients, families and palliative care clinicians actually talk about prognoses in the natural decision-making setting. Understanding these existing patterns of communication is essential to identify the approaches that promote preference-concordant treatment decisions and enhance quality of life. The purpose of the PCCRI is to understand the context, content, process, and outcomes of prognosis communication as occurs naturally during palliative care consultations. Because existing models of prognosis communication are in early evolution [15, 28], the PCCRI uses a mixed-methods approach that measures features of prognosis conversations having *a priori* conceptual importance (quantitative component) and examines interactions ethnographically (qualitative component) to expand our existing communication frameworks. Here, we describe the study design; observation and measurement methods; and recruitment experience during the first year of PCCRI enrollment.

## Methods

### Design overview

The PCCRI is a multi-site observational cohort study of inpatient palliative care consultations as they naturally occur in the hospital setting. We restricted this study to the hospital setting because palliative care consultation in the outpatient context is comparatively newer, less frequent and highly variable in terms of reasons for referral [29, 30]. All participating patients or their healthcare proxies complete a brief pre-consultation (18 items) and post-consultation (2 items) interviewer-administered questionnaire. The initial consult visit and up to two additional subsequent visits are audio-recorded and directly observed using field observation methods. Up to 60 patient/proxy participants will complete an optional extended interview regarding their experience of illness and with the palliative care consultation. All participating palliative care clinicians complete a 35-item questionnaire about themselves at the time of enrollment and a 3-item questionnaire after each consultation that is about the patient's prognosis, performance status and goals of medical treatments. Patients’ medical and administrative records are collected at baseline, 1-month and 6-months.

### Conceptual foundation

Promoting patient-centered care in serious illness is a major goal for healthcare in the 21^st^ century [31–33]. Patient-centered care considers the patient’s unique experience of illness on shared ground with the clinician’s perspective. Patient-centered care directs clinicians to see the world both through the patient’s eyes and through a clinical lens, and promotes meaningful decisions that reflect each patient’s unique clinical context, personal values and preferences [34–36].

Our conceptual model of Patient-Centered Prognosis Communication [15, 20] is based upon prior work by Epstein and Street [37, 38] and identifies four specific and observable domains: 1) participants ENGAGING in discussion about prognosis, including inviting discussion of prognosis, eliciting preferences for information and respecting opinions; 2) RESPONDING to presence of emotion; 3) patient/family/clinician mutually INFORMING about prognosis opinions and perspectives, and 4) FRAMING uncertainty. Each of these overlapping and mutually influenced domains assumes greater or lesser importance depending on the clinical context. We define “prognosis communication” to include prediction or anticipatory guidance about illness course, including how treatment options might influence such prognoses.

Measuring patient-centered communication can focus on process and outcomes [36, 39]. We do both. Wide agreement exists that good communication should promote patients feeling heard and understood and, ultimately, match medical care to the patient’s unique context, preferences and personal values—we consider these to be patient-centered *outcomes* [32, 36, 37, 39]. However, process measures are less developed, largely because few clinical situations are understood empirically well enough to know *a priori* how theoretically-driven behaviors are organized to promote desired outcomes [36, 39]. This research considers conversations as important *processes* of patient-centered care.

### Clinical setting

#### The University of Rochester Medical Center (URMC) Inpatient Palliative Care Services

URMC is comprised of a 750-bed academic medical center and a 261-bed community hospital, both located in the city of Rochester, NY. The URMC PC inpatient consultation service cares for over 1,600 patients per year --about half of whom have advanced cancer--and is staffed by 24 attending physicians (rotating on 2-week blocks), 3 fellows (rotating on 4-week blocks) and 5 nurse practitioners (continuous).

#### University of California at San Francisco (UCSF) Inpatient Palliative Care Services

UCSF’s Moffitt-Long Hospital is a 600-bed academic medical center located in the city of San Francisco, CA. The UCSF PC inpatient consultation service cares for over 800 patients per year --about half of whom have advanced cancer--and is staffed by 9 attending physicians (rotating on 1-week blocks), 4 physician fellows (rotating on 1-month blocks), as well as 2 social workers, 1 clinical nurse specialist, and 2 chaplains (all continuous).

### Participants

#### Clinician participants

All palliative care team clinicians are eligible to participate, including attending and fellow physicians, nurse practitioners, social workers, and spiritual care providers. Trainees rotating briefly with the palliative care team and clinicians working with other services in the hospital may be present at the time of audio recording (*eg*. resident physicians, medical students, bedside nurses, unit social workers, etc.), but their names are removed from the audio recording.

#### Patient (and healthcare proxy) participants

All English-speaking adult patients who are referred to palliative care and whose primary life-limiting illness is a metastatic cancer are eligible for this study. We define “solid cancers” to include all non-hematologic malignancies and lymphomas. We define “metastatic” to include Stage 3 or 4 cancers or, if staging is incomplete, the oncologist’s clinical judgment about the presence of distant metastases from a known primary cancer. We exclude patients who are receiving hospice care or have a “Comfort Measures Only” designation in their Medical Orders for Life Sustaining Treatments (MOSLT) at the time of referral because this study is focused on consultations where active decision-making about disease-oriented treatments is relevant. If potentially eligible patients lack basic decision-making capacity (per clinical team determination), then the surrogate decision-makers are eligible to participate. Surrogates are eligible if they speak English and have assumed official Health Care Proxy status, either by state law or patient assignment. Therefore, we refer to surrogates in this study as "healthcare proxies".

### Recruitment and enrollment

The sustained success of this project rests on the ability to identify, approach, inform, consent and enroll participants amid the usual workflow of the inpatient palliative care consultation service. During a two-year preliminary study funded by the National Palliative Care Research Center [20–22, 24] as well as the pilot phase of the PCCRI, we developed and refined the following protocol with close collaboration of the clinical palliative care team:

#### Preliminary identification

When a patient is referred for palliative care consultation, the accepting clinical provider identifies potentially eligible patients using a PCCRI pocket eligibility card as needed for reference. PCCRI Study Staff assist clinicians in determining eligibility questions or clarifications.

#### Initial approach

**Fig. 1:**
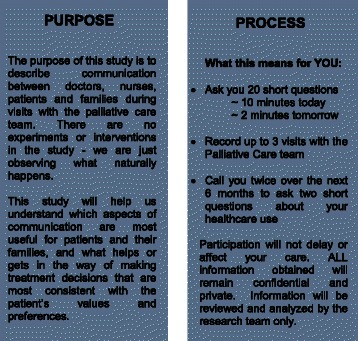
PCCRI Introductory Informational Brochure*. *for space, removed brochure flaps containing research contacts and Human Subjects Review Board approvals

After the patient or family has met a member of the clinical palliative care team, eligible patients or family members are asked by a member of the clinical team (URMC), or the study coordinator (UCSF) whether they would be willing to hear more about the study. The PCCRI Study Brochure is used to provide information about the study (see Fig. 1). Patient or proxies who agree to hear more about the study are given detailed information as consistent with informed consent procedures by the PCCRI Study Staff in their hospital room. Patients/proxies who agree to participate sign written informed consent.

#### Remuneration

Participating patients/proxies receive a total of $20 for participation ($10 after each pre-post consultation questionnaire). Participating palliative care clinicians receive $100 at the time of baseline questionnaire.

The study research coordinator documents all recruitment efforts regarding eligibility, participation, and the reasons for ineligibility, opting out or non-participation (if reasons are offered).

### Sampling approach

At each study site, 0–2 consultations with potentially eligible patients typically occur each day. However, there are days when substantially more eligible consultations happen. We approach potentially eligible patients in the order that referrals occurred during the day, with the expectation of enrolling up to two on any given day (depending on staffing). When potentially eligible consultations co-occur, we prioritize approaching patients from racial or ethnic backgrounds that are historically underrepresented in healthcare research.

### Data collection & measurement instruments

We designed the PCCRI to understand the relation between clinical communication and patient-centered outcomes (see Conceptual Foundation). In order to validly accomplish this aim, the PCCRI includes self-report (*eg*. patient perceptions & preferences; clinician perceptions), direct observation & audio recording of palliative care consultations and medical record abstraction (*eg*. disease status; treatment utilization). The PCCRI study data are collected from eight sources as described below: (1) hospital medical & administrative record; (2) patient/proxy baseline questionnaire; (3) palliative care clinician baseline questionnaire; (4) digital recordings of the palliative care conversations; (5) direct observation checklist & field notes of the palliative care conversations; (6) patient/proxy post-consultation questionnaire; (7) palliative care clinician post-consultation questionnaire; and (8) an in depth post-consultation interview among a sub-sample of patient/proxy participants. “Post-consultation” here refers to the next calendar day following the first recorded conversation with the palliative care team, regardless of whether the palliative care team continues to care for the patient during the remaining hospitalization.

#### Hospital medical & administrative record

The research assistant identifies healthcare utilization and the site of death of each study participant *via* 1-month and 6-month interval telephone contact with health care provider or family member contact provided by the participant at time of enrollment. For any hospitalizations or hospice enrollment, we extract pertinent treatment data (see outcomes) *via* a standard health services utilization form used in related palliative care health services research [40, 41].

#### Patient/Proxy baseline questionnaire

Following consent procedures, each patient or proxy participant completes an 18-item interviewer-administered questionnaire. The questionnaire is purposefully designed to be of very low time and cognitive burden in order to promote a representative participant sample amid the often stressful context of palliative care consultation. As such, we modeled most questionnaire items based on the Dartmouth COOP Chart item and categorical response structures for their strong validity and reliability in the clinical setting [42–44]. This single-item approach promotes efficient and effective measurement of multiple constructs when sensitivity to change is not a primary methodological priority. We performed iterative cognitive interviewing and pilot testing for each questionnaire item and for the final instrument to ensure question comprehension, appropriate response options and minimal burden. The usual time for completion is fewer than 10 min. Table 1 shows the specific questions and response options in the order that they are asked. We identify the relevant constructs for each item and reference sources that we used in their original or modified form.

**Table 1:** Patient questionnaire measures

Question	Response	Construct	Source
^a^1. Considering all parts of your life – physical, emotional, social, spiritual, and financial – over the past two days, how would you rate your quality of life?	0-10 scale	QOL- Global	McGill Quality of Life Questionnaire, Global item [61, 62]
	Very Bad (0)		
	Excellent (10)		
2. Over the past two days, how much have you been bothered by physical problems such as pain, upset stomach or difficulty breathing?	Not at all	QOL- Physical	Modified from Dartmouth COOP “Feelings” Chart [43]
	Slightly		
	Moderately		
	Quite a bit		
	Extremely		
3. Over the past two days, how much have you been bothered by emotional problems such as feeling anxious, depressed, irritable, or downhearted and blue?	Not at all	QOL- Emotional	Dartmouth COOP “Feelings” Chart [43]
	Slightly		
	Moderately		
	Quite a bit		
	Extremely		
4. Over the past two days, how much have you been bothered by uncertainty about what to expect from the course of your illness?	Not at all	QOL- Prognostic Uncertainty	Modified from Dartmouth COOP Chart (“Bothered by”) [43] and “what to expect from the course of my illness” [63]
	Slightly		
	Moderately		
	Quite a bit		
	Extremely		
5. Over the past two days, how much have you felt at peace?	Completely	QOL- Spiritual	Modified from Peace Screening Item [64]
	Quite a bit		
	Moderately		
	Slightly		
	Not at all		
^a^6. Over the past two days, how much have you felt heard and understood by the doctors, nurses and hospital staff?	Completely	Perceptions of Patient-Centered Communication	Modified from the Healthcare Climate Questionnaire [65] and the Patient Perceptions of Patient-Centeredness Questionnaire [36, 66]
	Quite a bit		
	Moderately		
	Slightly		
	Not at all		
7. – 10. Gender (including transgendered), Ethnicity, Race, Education			
11. When you think about the amount of income that you have available in a typical month, how often is it enough for things you really need like food, clothing, medicine, repairs to the home, and transportation?	All of the time	Financial Strain	Modified from VOICE Study [49]
	Most of the time		
	Some of the time		
	None of the time		
12. How well does the following statement describe you? “In uncertain times, I tend to expect the best.”	Completely	Dispositional Optimism	Life Orientation Test, main item [67, 68]
	Quite a bit		
	Moderately		
	Slightly		
	Not at all		
13. What religions, if any, do you maintain a connection with? (Please check all that apply)	Christianity, Judaism, Islam, Hinduism, Buddhism, Other, No formal religious affiliation	Identity with Religion	VOICE Study [49]
14. How much are your spiritual needs being supported by a religious community (like clergy or members of a congregation)?	Completely	Spiritual Support- Religious	Coping with Cancer Study [69]
	Quite a bit		
	Moderately		
	Slightly		
	Not at all		
15. How much are your spiritual needs being supported by the medical system (doctors, nurses and chaplains)?	Completely	Spiritual Support- Medical	Coping with Cancer Study [69]
	Quite a bit		
	Moderately		
	Slightly		
	Not at all		
16. How would you describe the purpose of your current medical care? Would you say	Yes	Perceptions of Treatment Purpose	Temel Study [23]
	No		
	Don’t know		
A. “to help you live longer?”	(for each a-c)		
B. “to help you feel better?”			
C. “to get rid of all the cancer?”			
17. Would you say that it is likely or unlikely that you will live for a year or longer?	Very Likely	Self-Reported Life Expectancy	Adapted from Diefenback [70]
	Likely		
	Unlikely		
	Very Unlikely		
	No idea		
18. How strongly do you agree or disagree with the following statement?	Strongly Agree	End of Life Treatment Preferences	Modified from SUPPORT Study [1, 71]
	Somewhat Agree		
	Not sure		
“During the last few months of my life, I would prefer a plan of treatment that focused on my comfort and quality of life, even if that meant not living quite as long.“	Somewhat Disagree		
	Strongly Disagree		

^a^ asked at baseline questionnaire and on the calendar day following the initial recorded conversation, changing “Over the last 2 days…” to “Today…” on the latter assessment

#### Palliative care clinician baseline questionnaire

Each participating palliative care clinician completes a self-administered baseline questionnaire once at the time of consent. The questionnaire takes fewer than 20 min to complete and collects information about age, gender, ethnicity/race, religious affiliation, professional training, clinical practice type, and clinical experience. Given our emerging understanding of clinician mindfulness in fostering patient-centered communication [45], we include a shortened version of the Mindfulness Attention Awareness Scale [46] that included the following 5-items about situational attention: “I find it difficult to stay focused on what’s happening in the present.”; “I forget a person’s name almost as soon as I’ve been told it for the first time.”; “It seems I am “running on automatic,” without much awareness of what I’m doing.”; “I find myself listening to someone with one ear, doing something else at the same time.”; “I find myself preoccupied with the future or the past.” Response options included the following 6 categories: “Almost Always”; “Very Frequently”; “Somewhat Frequently”; “Somewhat Infrequently”; “Very Infrequently”; “Almost Never”. Clinicians were also asked the following two open ended questions about perceived needs: “When thinking about having conversations with patients and families about ‘what to expect’, are there things that you would find helpful to prepare you, the team or the patient for such conversations?” and “When thinking about having conversations with patients and families about matching treatment options to their values and preferences, are there things that you would find helpful to prepare you, the team or the patient for such conversations?”

#### Digital audio recordings

We observe and digitally record consultations with consenting participants. After obtaining written informed consent, the study research assistant places a hand-held recorder with a built-in multi-directional microphone in an unobtrusive location in the hospital room (*eg*. bedside tray table). Prior to entry of the palliative care team, the study research assistant initiates the recording and returns after the visit to stop the recording. (Participants are shown how to stop the recorder if they wish to do so at any time during the visit.) Our approach yields high fidelity recordings that allow the coder to hear even weak voices amid clinical background noises, such as high flow oxygen, intravenous fluid pumps and heart rate/respiratory rate monitors.

#### Direct observation checklist and field notes

When space in the hospital room allows, the study research assistant will observe the conversation from a distance that is outside the immediate conversational space. All research assistants have clinical experience in the hospital environment (*eg.* nursing, speech pathology) and undergo training in field observation methods. The observer role is not to be a participant, nor is it to be completely inanimate. Instead, observers are trained to have a human presence without disturbing the natural clinical processes. For example, if an IV pump alarm is beeping during the conversation, the observer would not mute the sound (even though they know how to do this safely). If asked, observers kindly decline offering clinicians feedback or to recall any details about the conversation. However, if the patient asked the observer to pass them a cup of water from their tray or to help them find their nurse call button, then the observer would certainly do so if no clinical team member was available at the moment to help. We purposefully hire observers who have clinical experience in hospital settings and professional interest in research. Observers undergo weeks of practice prior to actual observation—and then weekly meetings thereafter—in order to navigate the nuance of being present but not disruptive. Observers meet weekly and ad hoc with each other and Study Investigators, as well, to provide supportive space for dealing with (and celebrating) the intensities of the human experience that they are present to observe (including suffering, joys) [47].

Immediately after the conversation, the research assistant observer completes a field note that includes a standardized direct observation checklist about the following environmental factors: number of persons in the room; proportion of people from clinical teams and proportion from family; hospital floor and room type; ambient noise and heat levels, type and intensity of ambient light; type and intensity of ambient smells; amount of foot traffic in/out of the room during the conversation; and whether the conversation contained any mention of expected survival time, fears of death/dying, artificial hydration or nutrition, or religious or spiritual beliefs. In addition, research observers qualitatively describe any noticeable barriers to the patient being able to speak during the conversation (*eg*. intubation, prolonged nebulizer treatment, BIPAP, extreme weakness, other) and their impressions of key non-verbal interactions or “moments” during the recorded conversation.

#### Patient/Proxy post-consultation questionnaire

On the day following the pre-consultation questionnaire and audio-recorded consultation, participants are asked two questions post-consultation that they were asked on the pre-consultation questionnaire: Global QOL (Item 1, Table 1) & Perception of Patient Centered Communication (Item 6, Table 1). We changed the time of reference in the root of each question to “today” from “the last 2 days” to avoid overlap in time period assessed on the pre-consultation questionnaire. These two items were selected because of their potential importance to understanding mediators of the association between palliative care communication and EOL treatment outcomes. We chose to wait until the following calendar day (instead of directly after the consultation) to allow opportunity for any initial treatment changes (*eg*. pain medications) or identified information needs (*eg*. details about a specific treatment option from the oncologist) arising form the recorded consultation to have occurred. Patients enrolled on a Friday completed post-consultation questionnaires on the following Monday. Date and time of completion are recorded for analytic purposes.

During pilot testing, we found that a total of 20 items (18 pre-consultation, 2 post consultation) represented an important threshold for patients/proxies as they considered study participation—more items were often perceived as overly burdensome. Additionally, we found that clinical condition of seriously ill patients often changed rapidly; the two selected post-consultation items were of very low burden cognitively and emotionally for participants to complete.

#### Palliative care clinician post-consultation questionnaire

Immediately after each audio-recorded consultation, the participating PC clinician completes a self-administered questionnaire consisting of the following 3-items about the patient’s condition and treatment:

- Treatment Goals: “How would you describe the current status of [Patient Name]’s decisions about their medical treatments after consulting with the PC team? (best guess)” Response options are based on the categories commonly recorded in Medical Orders for Life Sustaining Treatments documentation that palliative care providers use frequently: “No limitations to the types of medical treatments”; “Some limitations to the types of medical treatments”; “Comfort Measures Only approach to medical treatments”; and “Unknown or Undecided”.
- Survival Prognosis: “How would you describe [Patient Name]’s most likely survival time, assuming that their illnesses are allowed to take their natural course? (best guess)” Response options: “fewer than 24 h”; “days to fewer than 2 weeks”; “2 weeks to fewer than 3 months”; “3 months to fewer than 6 months”; “6 months or longer”. We chose these response options because these intervals are commonly used in clinical practice for establishing potential hospice (inpatient, outpatient) and comfort-care home eligibility in both Rochester and San Francisco areas.
- Functional Status: Palliative Performance Scale (PPS reference card provided) [48]

#### In-depth interview with patients/proxies post consultation (sub-sample)

Using a criterion-based sampling strategy, the research assistant invites selected patients/proxies to participate in an open-ended interview following the completion of the post-consultation questionnaire. The research assistants, who are trained in qualitative interviewing techniques, conduct a 20–60 min interview using open-ended questions (with probes) exploring the following domains: what patients/proxies find important to think about when making decisions about their current and future care; how the patient’s past experiences in health care may be influencing their current experience; recalled experiences that may help guide patients in making medical treatment decisions; the importance of religion and/or spirituality as they think about their healthcare choices; what they are expecting in the future; what may have surprised them about their meeting with the palliative care team; what parts of their palliative care conversations have been most important to them; and whether there were other things in the conversation that they might want to address.

### Conversation coding

We will use established methods to reliably code domains of Patient-Centered Prognosis Communication [15, 20, 21, 24] described in the Conceptual Foundation. We train two coders for approximately 30 h over a two-week period of time using a detailed codebook and example transcripts. The codebook includes precise definitions and examples of what should and should not be coded. After the initial training period, we double code 20 % of conversations distributed over the full coding period to identify and prevent drift in coder practice. Using this approach, we have demonstrated excellent inter-rater reliabilities for prognosis content (*kappa* >0.70) [20, 21].

Using professional transcriptions, coders evaluate each speaker turn in the conversation, referred to as a *segment*, for the presence of prognosis content. As described earlier, we define prognosis communication to include any prediction about the future course of illness, including symptoms, survival, cure or functional status. Coders will then sub-classify each occurrence of prognosis content as described in Table 2.

**Table 2:** Description of communication codes

Domain	Description/Examples
ENGAGING in discussion	
Initiator [20]	First speaker of prognosis content. Includes a question or statement.
Early onset [20]	Prognosis content occurring within first 5 min of conversation
Goal expression [72]	*My hope is that this treatment will help me to feel more energy and buy me some time so that I can go on a cruise this summer with my family.*
Mutually INFORMING	
Quantity [21]	Number of segments containing prognosis content
Topic [21]	
Cure	*It is very unlikely that my cancer will be cured.*
Survival	*I expect that you will live for days to weeks, rather than months to years.*
Function	*You will likely need more help getting around over the next few months…*
Symptoms	*Your shortness of breath is likely to worsen in the next weeks, and we can help…*
Conditional prognoses [20]	*If you choose to continue transfusions, you are likely to live a little longer and likely to spend much more time in the hospital.*
Goal-linked prognoses [22]	*One thing I hear that you are hoping for is to live until your son’s graduation; my sense is that there is a good chance that you will live that long….*
RESPONDING to Emotion^a^	
Type of emotion [24]	
Sadness	*I just don’t care what happens anymore [crying], I’m so alone.*
Fear/worry	*I’m so scared about what’s gonna happen to my family…*
Anger	*I don’t give a [expletive] what the [expletive] doctors say, I’m gonna beat this!*
Intense emotion [24]	Emotion interrupts speech pattern or emotion repeated within same segment
Compassion [24]	*I can see that this uncertainty is a burden on you and your family; I am committed to seeing you through this and have some ideas about how to help…*
FRAMING Uncertainty	
Affective cues [21]	
Optimistic	*The good news is I expect you will live for a few more months.*
Pessimistic	*Unfortunately, I expect you will only live for a few months.*
Deductive distance [21]	
Population	*About 30 % of people die within one month.*
Individual	*I believe that there is a 30 % chance you will die within one month.*
Ambiguous [73]	*Things ahead look pretty good.*
	*Your prognosis is poor but nobody can predict the future.*

^a^emotion that is identifiably about prognosis based on the language used within the statement or within the segment directly following another prognosis-containing segment

Given the early state of science related to prognosis communication, the individual PCCRI codes are agnostic with regards to what is considered “good” *a priori* (which differs slightly from related clinical trials [49]) Rather, our approach is purely descriptive in order to more broadly investigate the features of conversations that predict or mediate observed patient-centered outcomes. Because clinical conversations are dynamic and relational phenomena that are crafted by all participants, we endorse an ecological approach that considers the unit of analysis to be the conversation rather than the individual speaker.

### Analyses

#### Overview

This is a mixed methods study designed to understand the features of prognosis conversations that are associated with patient-centered outcomes. The study includes both epidemiological (quantitative) and ethnographic (qualitative) methods. Below, we describe each separately and identify how they inform one another.

#### Approach to epidemiological analyses

We will describe the frequency and distribution for each study variable. For descriptive analyses, we will examine the crude relation between conversation features and outcomes. For the purposes of sample size determination, we consider the specific association between conversation features and decision to pursue a comfort-oriented plan of care. For this outcome, we define a comfort-oriented plan of care as that which meets BOTH of the following considerations: 1) Within two-weeks of the index conversation, EITHER (a) enrollment in hospice or (b) a “Comfort Measures Only” plan of care documented in the patient’s Medical Orders for Life Sustaining Treatments (MOLST). The specific language used in the MOLST document is: “The patient is treated with dignity and respect. Reasonable measures are made to offer food and fluids by mouth. Medication, positioning, wound care, and other measures are used to relieve pain and suffering. Oxygen, suction and manual treatment of airway obstruction are used as needed for comfort.” and 2) No aggressive life-sustaining treatment following election of (a) or (b) above until death (or end of the 6-month follow-up period). We use the following three indicators of “aggressive life-sustaining treatment” as established by the Dartmouth Atlas Project as having little or no benefit to any patients with advanced cancer: endotracheal intubation, feeding tube placement or cardiopulmonary resuscitation [50]. In order to achieve standard hypothesis parameters (alpha = 0.05, beta = 0.20), we require 72 cases (with at least one control per case) to identify a difference of 0.5 SD in conversation features. In recognition that effect sizes are likely to be larger than this, we will remain cognizant of the likelihood that our study will be overpowered for some associations under study.

We will stratify the crude association for each conceptual domain on potential confounding variables, including the following: the patient’s race; age; gender; educational attainment; religious affiliation; insurance; marital status; household caregiver status; perceived social and family support; usual source of healthcare; health literacy; cancer type; symptom burden; functional status; physician estimate of survival prognosis; and decision-making capacity. Factors associated with the exposures and the outcome status, and influencing the crude estimate of association will be considered as potential confounders in adjusted analyses. We will adjust for confounding using a multiple conditional logistic regression model following an iterative model building procedure where covariates are retained in the model if their removal has a substantial influence on the observed magnitude of association (*i.e*. odds ratio).

In recognition that conversations are complex phenomena, we will conduct exploratory latent class analyses [51] to examine whether observed patterns among the individual domains (*i.e.* ENGAGING, RESPONDING, INFORMING, and FRAMING) suggest “clinical types” of conversations that are predictive of outcomes.

#### Concepts of mediation

We will examine the degree to which associations between conversation features and treatment outcomes are mediated by patients ratings of patient centered communication (feeling heard and understood) and by patient ratings of Quality of Life (McGill Global QOL measure). “Mediator” is equivalent to the epidemiological concept of “causal intermediate”. Thus, we are estimating the degree to which proximal communication outcomes mediate the relation between the quality of prognosis communication and preference-concordant end-of-life medial treatment. Potential mediators are assessed using the established measures that are collected *after* the recorded conversation and *before* the outcome assessment. Pre-post changes in the McGill Global Quality of Life item will be measured on a 0–10 continuous scale. Minimally Important Differences have yet to be empirically established; therefore, we will consider 1.0 SD to represent a clinically important difference. Pre-post change in the Patient Centered Communication item is categorical and will be analyzed as the percent “Completely” as shown effective for avoiding ceiling effects in medical communication settings [52].

Assessing causation—and particularly causal pathways—is challenging in non-experimental studies. Nonetheless, these mediating factors occur temporally between the initial conversation and treatment outcomes and are conceptually supported to be mediators of patient-centered care. Therefore, this study can provide important hypothesis-generating information for subsequent testing using experimental methods.

#### Measuring values and preferences for end-of-life care

Decisions about medical treatment in advanced cancer are preference-sensitive. We measure preferences at study outset, prior to the audio-recorded palliative care consultation. This is good timing to preserve temporality of the associations under study. However, stated preferences for treatments in such contexts are dynamic. When considering decisions that have never been faced before, people often start with preferences that are either uninformed or based on hypothetical situations. As people gather information, their preferences often change, and are ‘constructed’ based on a variety of personal, informational, affective and social factors [53, 54]. The instability in preferences, however, is not symmetric. People’s preferences are far more likely to change *from* aggressive cure-oriented treatments *toward* comfort oriented treatments than vice-versa [53]. Therefore, errors in assignment of preference-concordant EOL treatment are likely directional, that is, underestimation of concordance when comfort-oriented EOL treatment is found to occur. We will conduct sensitivity analyses to assess potential implications of this bias.

#### Approach to ethnographic analyses

Each interview and corresponding audio-recorded conversation is professionally transcribed verbatim, de-identified (replacing codes for spoken names) and checked for accuracy by PCCRI research assistant. The research assistant corrects any errors, and notes any extended pauses, emotional displays, or disruptions (*eg.* people leaving the room, telephone calls). The corrected transcript will be dated, timed, and entered into the Atlas.ti 7.2 data management software program. Audio files will also be imported into Atlas.ti. Atlas.ti 7.2 allows for the integration of textual and audio data, resulting in more efficient data management.

We will use a phased approach to analysis. In phase one, we will use an inductive open coding approach. During the second phase of coding, we will use our Conceptual Model as a set of sensitizing concepts—ideas believed to be important to a phenomenon [55]. In phase three, we will compare the findings from earlier phases by examining similarities, differences, gaps and areas of overlap. This analysis will strengthen our understanding of how patients' prior experiences and prognosis-related beliefs manifest within our existing conceptualizations of prognosis communication.

We will apply our developed coding scheme to characterize patterns of content, structure and process among the cases, using strategies developed by Saldana, Miles and Huberman [56].

#### Future analyses

The PCCRI is a comprehensive cohort study that includes recorded and transcribed conversations. As the science of communication continues to evolve, the PCCRI infrastructure presents extraordinary opportunities for further nested studies that analyze conversations for new content (*eg*. developing or evaluating new quality indicators for palliative care practice [57]) or new methods for measuring and understanding the complexities of human communication (*eg.* natural language processing computational linguistics [58]).

#### Ethics statement

This study is approved by the research review committees at the University of Rochester Medical Center and the University of San Francisco Medical Center. This manuscript adheres to the STROBE standards for reporting of observational studies. We grant permission to other scientists to use all study protocols and de-novo measurement items described here for non-commercial purposes.

## Results

### Patient-participant enrollment rate

The PCCRI began enrollment at the URMC site in January of 2014 and the UCSF site in March 2015. This report covers the URMC enrollment period of January 6, 2014 to February 8, 2015. During this period, a member of the clinical team invited 188 potentially-eligible patients to learn more about a palliative care communication research study based on the following initial screening criteria: cancer type and stage, not enrolled in hospice at time of referral, English speaking, decisional capacity or known proxy present. One hundred forty three agreed to hear more, forty-three declined and two expressed potential interest but were ultimately not approached due to simultaneous enrollment of other participants. Among the 143 who were then informed about the PCCRI by a member of the research team, twenty-three declined participation and two wished to participate but were seen by the palliative care team before informed consent procedures could be completed. One hundred fourteen patients (14 % by proxy) decided to participate and completed written informed consent. Therefore, our initial one-year enrollment rates are 61 % among those invited and 80 % among those informed. Reasons for non-participation were not solicited, however the most common spontaneously volunteered reasons were lack of energy, feeling overwhelmed and wishing to wait until family members arrive (which would occur after the enrollment window).

#### Patient-participant approach rate

As described above, 188 patients were invited to hear about a communication research study. Since the research screening process occurred by clinicians amid the processes of usual care, the PCCRI collects data only from patients who elect to participate. Therefore, the exact number of the eligible population is not known. Based on historical aggregate QI data from the URMC site, 45 % of new consultations are for patients with cancer and 51 % of this group had full capacity to make medical decisions. Applying these proportions to the observed 1,173 new patient consultations that occurred during the January 2014 and February 2015 initial period of PCCRI recruitment, 269 patients would have cancer and capacity to consent for participation. We have no data available to assess the prevalence of other eligibility criteria. However, we estimate that the only minimal net effects of these unknowns on the eligible population size based on judgment that the remaining exclusions (ie. English speaking, solid cancer, metastatic disease, and not enrolled in hospice at time of referral) would be offset by the number of incapacitated patients having a Health Care Proxy physically present at time of consultation to allow proxy-based participation. Therefore, we approximate an *Approach Rate* of 70 % (188 approached /269 eligible) and a population sampling proportion of 42 % (114 enrolled/ 269 eligible).

Black/African American participants represent 12 % of the initial PCCRI sample—a proportion nearly identical to that observed for the palliative care clinical service (2006–2010 historical data [21]). No patients/proxies have expressed concerns about being invited to participate and no participants have experienced research adverse events.

#### Clinician-participant enrollment rate

All palliative care clinicians who have been approached for potential participation (all clinicians at the URMC site and the initial ten at the UCSF site) enrolled in the PCCRI.

## Discussion

The Palliative Care Communication Research Initiative is a unique cohort study that integrates self-report (patient, proxy, clinician), direct observation (field ethnographic observation, audio-recording) and health services utilization data in order to understand the characteristics of clinical communication that promote patient-centered outcomes. Our experience during the first year of enrollment confirmed that such work is feasible, acceptable and sustainable in the natural clinical setting of palliative care.

Nearly two out of every three patients (or proxies) who were invited to participate chose to enroll in the PCCRI, thus promoting generalizability. No patients reported harms related to invitation or participation. We attribute this successful experience to four key factors. First, this study focuses on understanding communication—something that seriously ill patients and their families identify as in need of improvement [3, 18, 59]. We developed the PCCRI informational brochure to make this focus clear to potential participants (see Fig. 1). Anecdotally, many patients, proxies and family members spontaneously express gratitude for being able to contribute to the science of communication for future patients. The PCCRI mails participants (or their family contacts identified at study outset) *Thank You* and *Bereavement* cards as well as offering to notify participants of study findings when they become available.

Second, we minimized the burden of self-report by using brief measures specifically designed for ease of use and reliability the natural clinical setting [43, 44, 60]. The primary methodological trade-off is that using single item measures with stable categorical response options yield low sensitivity to change over time. Since most self-report measures are being used for assessing confounding, effect modification or mediation, we fully accept this trade-off to promote representative sampling. Furthermore, our pilot testing of the PCCRI protocol identified a total of 20 questions to be an important threshold that patients/proxies often weighed in their consideration of participation. We encourage other investigators to similarly evaluate such item threshold in their research context because we have found this single decision to have substantive effects on participation rates.

Third, the PCCRI observers all have substantial clinical experience in the hospital setting (eg. nursing, speech pathology) and are savvy about research methods. Most of the PCCRI observers have either completed or are completing graduate research degrees. This background allows the PCCRI observers to navigate the complexities of conducting high quality research amid the substantial and dynamic demands on the clinical team. They are adept at balancing supporting clinical efficiency and valid sampling/measurement. The PCCRI observers and the PC clinicians have developed a strong and genuine partnership that has created the energy necessary to sustain this type of work.

Fourth, and likely most important, is that the PCCRI is comprised of supportive and enthusiastic palliative care clinical teams. The PCCRI palliative care clinicians appreciate the need for communication research in serious illness and want to support these efforts. Indeed, 100 % decided to participate. Being observed and audio-recorded can be uncomfortable, especially in the intimate settings of inpatient palliative care consultation—participating in this work takes a level of trust in the investigative team. We have found three factors, in particular, offset this potential barrier to participation. First, and foremost, is that the PCCRI palliative care clinicians are comfortable with their skills and interested in promoting the science of patient-centered communication. Without this, we do not believe that this work would be feasible. Second, four study investigators (including study PI and site PI’s) are also physician-participants in the study, thus maintaining awareness of the study procedures on personal vulnerabilities and the overall clinical team functioning. (To protect against the introduction of bias, all analyses will evaluate whether excluding the investigator-participant data substantively influences the observed findings.) Third, the PCCRI espouses a descriptive and ecological approach to studying clinical communication rather than a prescriptive and hypercritical one. We consider the conversation as the unit-of-analysis and that these phenomena are crafted by all participants in clinical context. Our track record of publication and presentation demonstrates this objective approach that is void of editorializing or sensationalizing [20–22, 24]. Remuneration of the clinicians’ time for answering questionnaires is negligible and unlikely to have any effects on choices to participate.

This study has important limitations. First, our protocol does not attempt to record all interactions with the PC team. For example, we do not approach potential participants until they have at least had a chance to meet a member of the PC clinical team. Although this initial clinical PC visit is usually quite brief, our data might be missing communication that is important to decision-making and patient-centered care. Second, we limited patient/proxy self report to 20 items. Although we have found this decision to promote greater participation and representative sample, it did require us to abandon the systematic collection of other important data (*eg*. experience of social isolation/loneliness, existential QOL, experience of discrimination in the medical system). Third, the patient/proxy questionnaire is novel; it has not been used before. We partially address this limitation by adapting our individual questions and response options on previously validated items and by performing multiple pilot tests in our study setting for flow, burden and comprehension. Fourth, our follow-up of patient-reported outcomes is limited to 24 h; the remainder of the 6-month follow-up data is collected by medical record extraction and/or communication with patient-participants’ healthcare providers. This allows for valid and reliable determination of health services outcomes (*eg*. treatment, costs, survival and place of living/death) but not for QOL and bereavement outcomes. We will remain cognizant of these limitations in our interpretation and dissemination of PCCRI findings.

## Conclusion

In summary, early findings from the Palliative Care Communication Initiative support the feasibility, acceptability and sustainability of conducting direct-observation epidemiologic research in the natural setting of palliative care. Given that communication is the core “procedure” of palliative care, work of this kind is crucial to understand, support and disseminate best practices for this rapidly growing field of clinical care.

## Abbreviations

PCCRI: Palliative Care Communication Research Initiative
QOL: Quality of life
BIPAP: Bilevel positive airway pressure
URMC: University of Rochester Medical Center
UCSF: University of San Francisco
